# A comparison of zero-profile anchored spacer (ROI-C) and plate fixation in 2-level noncontiguous anterior cervical discectomy and fusion- a retrospective study

**DOI:** 10.1186/s12891-018-2033-7

**Published:** 2018-04-17

**Authors:** Zongyu Zhang, Yawei Li, Weimin Jiang

**Affiliations:** 1 Department of Orthopaedic Surgery, The First Affiliated Hospital of Soochow University, 899 Pinghai Road, Suzhou, China; 2 Department of Orthopaedic Surgery, Lianyungang Affiliated Hospital of Nanjing University of Chinese Medicine, 148 Chaoyang Road, Lianyungang, China

**Keywords:** Anterior cervical discectomy and fusion (ACDF), Cervical disk degenerative disease (CDDD), Two noncontiguous levels

## Abstract

**Background:**

Anterior cervical discectomy and fusion (ACDF) is the classic surgical treatment for symptomatic cervical degenerative disc disease (CDDD). However, there is controversy over the best surgical management in patients with two noncontiguous symptomatic levels of CDDD.

**Methods:**

From April 2011 to May 2014, 44 patients with two noncontiguous symptomatic levels of CDDD underwent skip-level ACDFs. In Group NoPlate, 23 cases underwent 2 noncontiguous levels of ACDF using zero-profile anchored spacer; and in Group Plate, 21 cases underwent 2 noncontiguous levels of ACDF using cages and plates. Operation-related paraeters for each group were recorded and compared. Japanese Orthopedic Association (JOA) scores and Neck Disability Index (NDI) scores at preoperation and postoperation were compared with at least a 2-year follow-up. Cervical lordosis was analyzed before surgery, 1 month after surgery, 3 months after surgery, and at final follow-up.

**Results:**

Mean follow-up was 35.4 ± 6.5 (range 24–48) months. Significant improvement on the JOA, NDI scores and cervical lordosis was noted in each group (*p* < 0.05), and there were no significant difference in terms of JOA, NDI scores, cervical lordosis and fusion rate between the two groups (*P* > 0.05). The operation time in Group NoPlate was significantly shorter than in Group Plate (*p* < 0.05), and the incidence of dysphagia and adjacent segment degeneration in Group NoPlate was significantly lower than in Group Plate (*p* < 0.05).

**Conclusions:**

ROI-C and cages with plate fixation were both effective in two-level noncontiguous ACDF, and there were no significant difference in clinical outcomes, fusion rate, and cervical lordosis. However, ROI-C was associated with shorter operative time, lower incidence of dysphagia and adjacent segment degeneration.

## Background

Anterior cervical discectomy and fusion (ACDF) was first reported in the 1950s [1], which then has become the classic surgical treatment for symptomatic cervical degenerative disc disease (CDDD) [2]. Many studies showed that single- and multilevel continuous ACDFs achieved good results [3–7]; however, there is controversy over the best surgical management in patients with two noncontiguous symptomatic levels of CDDD. Surgeons may choose to perform a 3-level ACDFs rather than just treat symptomatic levels for fear of significant additive stress on the intermediate intervertebral disc. However, 3-level anterior fusions are associated with increased rates of pseudarthrosis and poorer clinical outcomes than shorter constructs [8, 9].

**Fig. 1:**
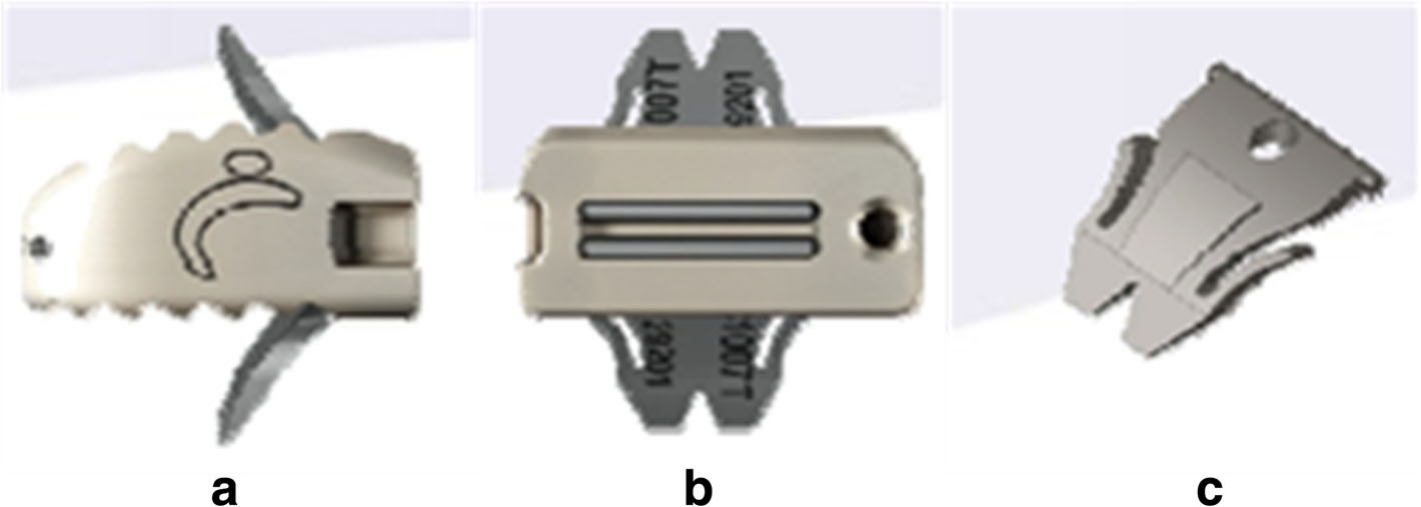
Lateral (**a**) and anteroposterior (**b**) views of the zero-profile anchored spacer (ROI-C, LDR, Troyes, France); (**c**) the integrated self-locking and self-directing clip

In 2-level noncontiguous ACDF, 2 plates are often used to provide the immediate postoperative stability and improve the fusion rate. However, the procedure of implanting plates is associated with the risk of perforation of esophagus and dysphagia. Besides, it may raise the risk of adjacent-level ossification [10, 11]. Previously, we investigated ACDF with zero-profile anchored spacer (ROI-C, LDR, Troyes, France) for the treatment of 1-level and 2-level contiguous CDDD, achieving satisfactory clinical and radiological outcomes [12]. This implant system is constructed of a polyether-ether-ketone (PEEK) cage and two integrated self-locking clips. The clips can enter the vertebral body through the endplate and provide anterior column fixation (Fig. 1). Two-level noncontiguous ACDFs with ROI-C, which only fuses the symptomatic levels without anterior plates, may therefore be the optimal treatment choice. The aim of the present study was to compare surgical parameters, clinical and radiological outcomes in patients who underwent 2-level noncontiguous ACDF with ROI-C or with cages and plates.

## Methods

### Patient population

From April 2011 to May 2014, a total of 44 patients underwent skip-level ACDFs.

Inclusion criteria were (1) symptomatic CDDD which was unresponsive to non-operative management; and (2) spinal cord or root compression at two noncontiguous intervertebral levels confirmed by magnetic resonance imaging (MRI). Exclusion criteria were: (1) significant instability of cervical spine; (2) developmental cervical spinal stenosis; (3) severe cervical deformity; (4) a previous history of cervical spine surgery; and (5) fracture, tumor, ossification of posterior longitudinal ligament and any serious general illness.

**Fig. 2:**
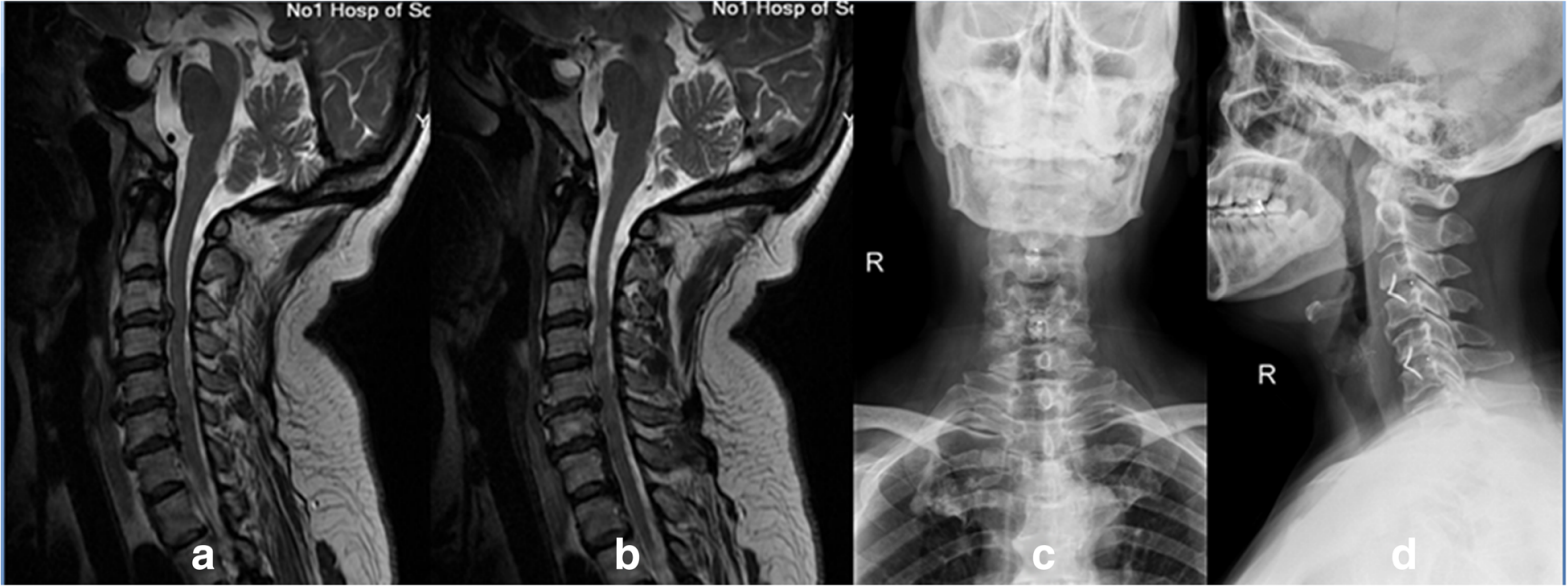
Preoperative lateral T2-weighted MRI (**a** and **b**) showing a 59-year-old woman with C3-C4 and C5-C6 disc herniation. Anteroposterior (**c**) and lateral (**d**) radiographs showing C3-C4 and C5-C6 anterior cervical discectomy and fusions (ACDF) with the zero-profile anchored spacers

**Fig. 3:**
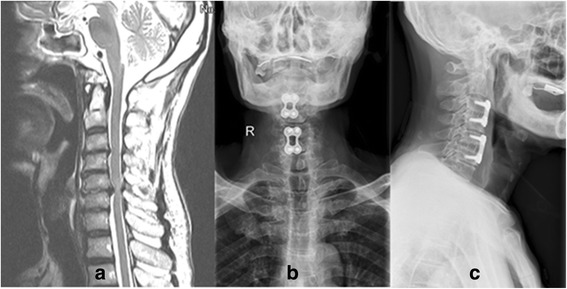
Preoperative lateral T2-weighted MRI (**a**) showing a 68-year-old man with C3-C4 and C5-C6 disc herniation. Anteroposterior (**b**) and lateral (**c**) radiographs showing C3-C4 and C5-C6 anterior cervical discectomy and fusions (ACDF) with cages and titanium plates

The 44 patients were divided into 2 groups: Group NoPlate (23 patients), who underwent fusion using ROI-C (Fig. 2); and Group Plate (21 patients), who underwent fusion using cages and plates (Medtronic, Minneapolis, American) (Fig. 3). There were no significant differences in the demographic data between the two groups (*P* > 0.05) (Table 1). This study was approved by the Institutional Ethics Committee of Soochow University.

**Table 1:** Demographic data of patients

	Group NoPlate	Group Plate	*P* value
Patients No	23	21	–
Gender (male: female)	15:8	9:12	0.137
Average age (years)	53.3 ± 8.8 (40–68)	57.8 ± 9.2 (39–70)	0.105
Operated levels	C3-C4 and C5-C6: 13; C4-C5 and C6-C7: 10	C3-C4 and C5-C6: 8; C4-C5 and C6-C7: 13	0.222
Blood loss (ml)	70.0 ± 15.8	75.4 ± 23.0	0.37
Operative time (min)	126.0 ± 13.2	143.4 ± 17.9	0.001
Follow-up period (months)	34.7 ± 7.6 (24–48)	36.2 ± 5.2 (26–48)	0.447

### Surgical technique

Operations were performed in supine position under general anesthesia using the standard right-sided anterior approach. The basic procedures including exposure, discectomy, and decompression were performed as described previously [1]. To achieve adequate decompression, the posterior longitudinal ligament, osteophytes, and other compressive elements should be removed after discectomy. Great care was taken to remove the cartilaginous tissue, but preserve the bony endplate to prevent cage subsidence. Cages were then implanted into the intervertebral space.

Zero-profile anchored spacers were used in Group NoPlate. In Group Plate, PEEK cages were inserted, and anterior plates were applied. In both groups, each appropriate-sized cage was packed with 0.25 mg of recombinant human bone morphogenetic protein (rhBMP-2, pharmaceutical group investment limited corporation, Hangzhou, China) and excised local osteophytes.

Patients were allowed to sit up on the first day postoperatively and walk on the second day postoperatively with a Philadelphia neck collar, which was applied for 4 weeks.

### Collected data and outcome assessment

Collected data included age, gender, operated segments, intraoperative blood loss, operative time, complications, and clinical and radiologic outcomes. The complete cervical spine X-ray, computed tomography (CT), and magnetic resonance imaging (MRI) were performed preoperatively. After surgery, the cervical spine X-ray were routinely taken. Clinical and radiological evaluations were performed preoperatively (1 day before operation) and postoperatively at 2 weeks, 1 month, 3 months, and the final follow-up. Radiological outcomes were measured using the Picture Archiving and Communication System (PACS) imaging system.

The Japanese Orthopaedic Association (JOA; scored from 0 to 17, with a lower score indicating more severe symptoms) scoring system was used to evaluate the neurological status [13]. Neck Disability Index (NDI; scored from 0 to 100%, with a lower score indicating less severe symptoms) scoring system was used to assess the neck function [14]. The JOA recovery rate (RR) was calculated using the rationale of Hirabayashi [15]: (postoperative score - preoperative score)/(17 - preoperative score) × 100. The incidence of dysphagia was assessed using the Bazaz grading system [16]. Severity of dysphagia was graded as none, mild, moderate, or severe (Table 2).

**Table 2:** Bazaz grading system for dysphagia

Symptom severity	Liquid food	Solid food
None	None	None
Mild	None	Rare
Moderate	None or rare	Occasionally (only with specific food)
Severe	None or rare	Frequent (majority of solids)

**Fig. 4:**
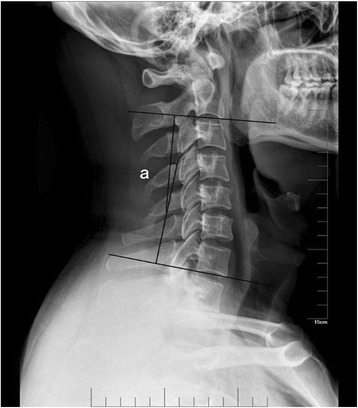
Lateral radiograph showing cervical lordosis (Cobb angle of C2–C7) calculated by measuring the angle formed by the lines along the inferior endplate of C2 to the inferior endplate of C7 in the neutral position

X-ray and CT scan reconstructions were used to evaluate the bony fusion. Satisfactory fusion include the following conditions [17]: (1) no motion between the spinous processes; (2) no radiolucent gap between the graft and the endplates; (3) continuous bridging bony trabeculae at the graft-endplate interface. The cervical lordosis was assessed by measuring the Cobb angle of C2–C7 from the inferior endplate of C2, to the inferior endplate of C7 in a neutral position (Fig. 4). The radiological evidence of adjacent segment (above, below and intermediate) degeneration included the following radiological manifestations: new osteophyte or enlargement of previous osteophyte; narrowing of a disc space; and increased calcification of anterior longitudinal ligament. Clinical and radiological assessments were conducted and reviewed by two senior spine surgeons and patients were followed up for at least 24 months postoperatively.

### Statistical analysis

Data are shown as mean ± standard deviation. Intergroup comparisons were made using *t* test or Chi-square test (Fisher’s exact test).The paired data were compared with a paired sample t test. All the analyses were performed using the Statistical Package for the Social Sciences (version 13.0 SPSS, Chicago, IL, USA) and the difference was considered statistically significant at the *P* < 0.05 level.

## Results

### Surgical parameters

The operated noncontiguous levels were C3–4 and C5–6 (Group NoPlate, *n* = 13; Group Plate, *n* = 8), and C4–5 and C6–7 (Group NoPlate, *n* = 10; Group Plate, *n* = 13). Group NoPlate had a mean blood loss of 70.0 ± 15.8 ml and the average operative time was 126.0 ± 13.2 min. Group Plate had a mean blood loss of 75.4 ± 23.0 ml and the average operative time was 143.4 ± 17.9 min. No significant difference existed in age, gender, operated levels and blood loss between the 2 groups (Table 1). However, significant differences existed in operative time between the 2 groups (*P* < 0.05).

### Clinical outcome

**Fig. 5:**
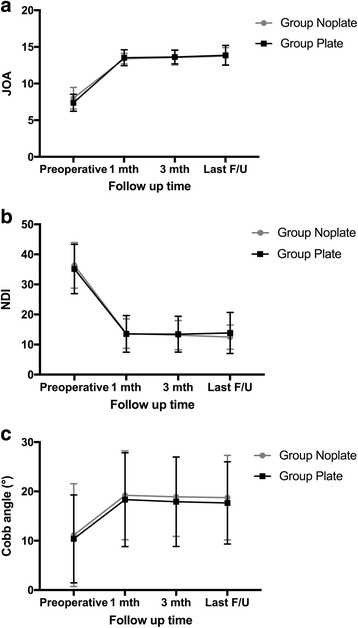
Line graphs showing a comparison of clinical and radiological results between Group NoPlate and Group Plate. Change trend of the Japanese Orthopaedic Association (JOA) score (**a**); Change trend of the Neck Disability Index (NDI) score (**b**); Change trend of cervical lordosis (**c**)

Patients were followed up from 24 to 48 (mean: 35.4 ± 6.5) months. In both groups, the JOA and NDI scores were significantly improved postoperatively (*P* < 0.01, Fig. 5a and b, Table 3). The comparisons of JOA and NDI scores at each follow-up time between the 2 groups showed no statistically significant differences (*P* > 0.05). The mean JOA RR was (62.6 ± 15.1)% in Group NoPlate and (66.8 ± 14.6)% in Group Plate, which was not statistically different (*P* > 0.05).

**Table 3:** Clinical and radiologic data evaluated before surgery and during follow-up

	Group NoPlate		Group Plate	
		*P* value^a^		*P* value^b^
JOA scores				
Preoperatvie	8.0 ± 1.5	–	7.4 ± 1.2	–
Postoperative 1 month	13.4 ± 0.7	< 0.001	13.5 ± 1.1	0.001
Postoperative 3 month	13.6 ± 1.0	< 0.001	13.6 ± 0.9	0.002
Last follow-up	13.7 ± 1.2	< 0.001	13.9 ± 1.4	0.001
NDI scores				
Preoperatvie	36.3 ± 7.6	–	35.1 ± 8.2	–
Postoperative 1 month	13.7 ± 5.3	< 0.001	13.5 ± 6.1	< 0.001
Postoperative 3 month	13.1 ± 4.9	< 0.001	13.4 ± 6.0	< 0.001
Last follow-up	12.5 ± 4.0	< 0.001	13.9 ± 6.8	< 0.001
Cervical lordosis (°)				
Preoperatvie	11.1 ± 10.4	–	10.4 ± 8.9	–
Postoperative 1 month	19.2 ± 9.0	< 0.001	18.3 ± 9.7	0.001
Postoperative 3 month	18.9 ± 8.0	< 0.001	17.9 ± 9.3	0.002
Last follow-up	18.7 ± 8.6	0.001	17.7 ± 8.3	0.001

^a^*P* value is given for comparison between pre-operative and post-operative values in Group NoPlate

^b^*P* value is given for comparison between pre-operative and post-operative values in Group Plate

### Radiologic outcomes

The cervical lordosis was significantly corrected postoperatively and the correction was maintained at last follow-up in both groups (*P* < 0.01, Fig. 5c, Table 3). No significant difference in cervical lordosis was found between the two groups at each follow-up time (*P* > 0.05). The fusion rate at 3 months postoperatively was 91.3% (21/23) in Group NoPlate and 95.2% (20/21) in Group Plate, which was not significantly different (*P* > 0.05). All patients achieved solid fusion at final follow-up.

### Complications

All patients tolerated the procedure well and no death was happened during the period of follow-up. No patients complained about dysphagia before operation. In Group NoPlate, 5 patients complained of dysphagia (2 moderate and 3 mild) on 2 weeks postoperatively, 3 patients had dysphagia (1 moderate and 2 mild) 1 month postoperatively, and no patients had dysphagia 3 months after the operation. In Group Plate, 12 patients complained of dysphagia (2 severe, 6 moderate and 4 mild) on 2 weeks postoperatively, 9 patients had dysphagia (4 moderate and 5 mild) 1 month postoperatively, 5 patients had dyphagia (1 moderate and 4 mild) 3 months postoperatively, and 4 patients still suffered from mild dyphagia at the last follow-up. There were significant differences in the presence of dysphagia between the two groups at all follow-up time points (Table 4). There were no adjacent segment degeneration observed in Group NoPlate and 19.0% (4/21) of patients had adjacent segment degeneration in Group Plate (3 patients developed adjacent-level ossification, and 1 patient developed intermediate-level disc space narrowing), and this difference was statistically significant (*P* < 0.05, Table 4).

**Table 4:** Complications after operations in the two groups

	Group NoPlate	Group Plate	*P* value
Dysphagia rate			
Postoperative 2 weeks	5/23	12/21	0.029
Postoperative 1 month	3/23	9/21	0.042
Postoperative 3 months	0/23	5/21	0.019
Final follow-up	0/23	4/21	0.044
Adjacent segment degeneration	0/23	4/21	0.044

## Discussion

The treatment of two noncontiguous symptomatic levels of CDDD poses a dilemma for spine surgeons. The surgical procedures commonly include anterior cervical corpectomy and fusion (ACCF), 3-level ACDF and 2-level noncontiguous ACDF. Compared with ACDF, ACCF has more bleeding, higher incidences of postoperative complications, and less improvements of cervical lordosis. Three-level ACDF sacrifices an additional motion segment, and has been associated with increased rates of pseudarthrosis and poorer clinical outcomes than shorter constructs [8, 9]. Finn et al. [18], in a cadaveric study, found that the adjacent levels beared a remarkable increase in stress in the three-level ACDF. However, in the two-level ACDF, the adjacent segments (above, below and intermediate) experienced modest stress relative to intact. Therefore, 2-level noncontiguous ACDF may be the optimal treatment choice.

There is controversy over the use of anterior plate in a single-level ACDF. However, it is beneficial to implant anterior plates in a multilevel ACDF [19], for the fusion rates were reported bo be unacceptably low after multilevel ACDF without plating [8, 20]. To provide immediate postoperative stability and increase the fusion rate, two plates are often applied in 2-level noncontiguous ACDF. However, the procedure of implanting plates at the optional position is very time consuming. On the contrary, the zero-profile anchored spacer, which has two integrated self-locking and self-directing clips, is simple to implant. In our study, we found the zero-profile anchored spacer was significantly superior to the anterior plate in terms of operative time.

Dysphagia is reported to be the most common postoperative complication after ACDF [21]. In most patients, dysphagia disappears within 3 months. However, there are 12.5–35.1% of cases still have dysphagia 3 months after the operation [16]. In our study, the presence of dysphagia in Group NoPlate is lower compared with that in Group Plate at all follow-up time points, and the duration of dysphagia is shorter. Although the mechanism of dysphagia remains unknown, the irritation of the esophagus by the anterior plate is considered to be a possible cause [16, 22, 23]. At the early stage (2 weeks postoperatively), it could also partly be attributed to longer intraoperative esophagus retraction time and greater retraction extent to fix the anterior plates. The zero-profile anchored spacer can be completely contained in the intervertebral space, avoiding the mechanical irritation of the soft tissue, especially the esophagus, resulting in lower incidence of postoperative dysphagia.

It is reported that the presence of anterior plates increased the incidence of adjacent segment degeneration [24]. Park et al. [11] found that an anterior cervical plate close to the adjacent intervertebral disc may cause adjacent level disc degeneration or surrounding bone formation. In our study, 4 patients in Group Plate developed adjacent segment degeneration, which was significantly higher than that in Group NoPlate. This may occur because the zero-profile anchored spacer can be completely contained in the intervertebral space, minimizing the irritation of the adjacent cervical structures.

This study has several limitations. First, it was retrospective. Second, the number of patients was small. Third, the follow-up period was relatively short. Finally, the final follow-up time was not consistent, which caused variation in adjacent segment degeneration. Thus, the results of this study need to be confirmed by large sample, prospective, randomized studies with long-term follow-up.

## Conclusion

ROI-C and cages with plate fixation were both effective in two-level noncontiguous ACDF, and there were no significant difference in clinical outcomes, fusion rate, and cervical lordosis. However, ROI-C was associated with shorter operative time, lower incidence of dysphagia and adjacent segment degeneration.

## Abbreviations

ACCF: anterior cervical corpectomy and fusion
ACDF: Anterior cervical discectomy and fusion
CDDD: Cervical disk degenerative disease
CT: Computed tomography
JOA: Japanese Orthopedic Association
MRI: Magnetic resonance imaging
NDI: Neck Disability Index

## References

[1] Smith GW, Robinson RA (1958). The treatment of certain cervical-spine disorders by anterior removal of the intervertebral disc and interbody fusion. J Bone Joint Surg Am.

[2] Gore DR, Sepic SB (1984). Anterior cervical fusion for degenerated or protruded discs. A review of one hundred forty-six patients. Spine.

[3] Papadopoulos EC, Huang RC, Girardi FP, Synnott K, Cammisa FP (2006). Three-level anterior cervical discectomy and fusion with plate fixation: radiographic and clinical results. Spine.

[4] Bolesta MJ, Rechtine GR, Chrin AM (2000). Three- and four-level anterior cervical discectomy and fusion with plate fixation: a prospective study. Spine.

[5] Chang SW, Kakarla UK, Maughan PH, DeSanto J, Fox D, Theodore N (2010). Four-level anterior cervical discectomy and fusion with plate fixation: radiographic and clinical results. Neurosurg.

[6] Kao FC, Niu CC, Chen LH, Lai PL, Chen WJ. Maintenance of interbody space in one- and two-level anterior cervical interbody fusion: comparison of the effectiveness of autograft, allograft, and cage. Clin Orthop Relat Res. 2005;(430):108–16. 10.1097/01.blo.0000142626.90278.9e 15662311

[7] Kim SW, Limson MA, Kim SB, Arbatin JJ, Chang KY, Park MS (2009). Comparison of radiographic changes after ACDF versus Bryan disc arthroplasty in single and bi-level cases. Eur Spine J.

[8] Emery SE, Fisher JR, Bohlman HH (1997). Three-level anterior cervical discectomy and fusion: radiographic and clinical results. Spine.

[9] Fraser JF, Hartl R (2007). Anterior approaches to fusion of the cervical spine: a metaanalysis of fusion rates. J Neurosurg Spine.

[10] Kim HJ, Kelly MP, Ely CG, Dettori JR, Riew KD (2012). The risk of adjacent-level ossification development after surgery in the cervical spine: are there factors that affect the risk? A systematic review. Spine.

[11] Park JB, Cho YS, Riew KD (2005). Development of adjacent-level ossification in patients with an anterior cervical plate. J Bone Joint Surg Am.

[12] Wang Z, Jiang W, Li X, Wang H, Shi J, Chen J (2015). The application of zero-profile anchored spacer in anterior cervical discectomy and fusion. Eur Spine J.

[13] Yonenobu K, Abumi K, Nagata K, Taketomi E, Ueyama K (2001). Interobserver and intraobserver reliability of the japanese orthopaedic association scoring system for evaluation of cervical compression myelopathy. Spine.

[14] Vernon H, Mior S (1991). The neck disability index: a study of reliability and validity. J Manip Physiol Ther.

[15] Hirabayashi K, Miyakawa J, Satomi K, Maruyama T, Wakano K (1981). Operative results and postoperative progression of ossification among patients with ossification of cervical posterior longitudinal ligament. Spine.

[16] Bazaz R, Lee MJ, Yoo JU (2002). Incidence of dysphagia after anterior cervical spine surgery: a prospective study. Spine.

[17] Hacker RJ, Cauthen JC, Gilbert TJ, Griffith SL (2000). A prospective randomized multicenter clinical evaluation of an anterior cervical fusion cage. Spine.

[18] Finn MA, Samuelson MM, Bishop F, Bachus KN, Brodke DS (2011). Two-level noncontiguous versus three-level anterior cervical discectomy and fusion: a biomechanical comparison. Spine.

[19] Resnick DK, Trost GR (2007). Use of ventral plates for cervical arthrodesis. Neurosurg.

[20] Bohlman HH, Emery SE, Goodfellow DB, Jones PK (1993). Robinson anterior cervical discectomy and arthrodesis for cervical radiculopathy. Long-term follow-up of one hundred and twenty-two patients. J Bone Joint Surg Am.

[21] Riley LH, Skolasky RL, Albert TJ, Vaccaro AR, Heller JG (2005). Dysphagia after anterior cervical decompression and fusion: prevalence and risk factors from a longitudinal cohort study. Spine.

[22] Lee MJ, Bazaz R, Furey CG, Yoo J (2005). Influence of anterior cervical plate design on Dysphagia: a 2-year prospective longitudinal follow-up study. J Spinal Disord Tech.

[23] Scholz M, Schnake KJ, Pingel A, Hoffmann R, Kandziora F (2011). A new zero-profile implant for stand-alone anterior cervical interbody fusion. Clin Orthop Relat Res.

[24] Chung JY, Kim SK, Jung ST, Lee KB (2014). Clinical adjacent-segment pathology after anterior cervical discectomy and fusion: results after a minimum of 10-year follow-up. Spine J Off J North Am Spine Soc.

